# Reduced Gaussian Kernel Filtered-x LMS Algorithm with Historical Error Correction for Nonlinear Active Noise Control

**DOI:** 10.3390/e26121010

**Published:** 2024-11-22

**Authors:** Jinhua Ku, Hongyu Han, Weixi Zhou, Hong Wang, Sheng Zhang

**Affiliations:** 1 College of Computer Science, Sichuan Normal University, Chengdu 610101, China; jhku@stu.sicnu.edu.cn (J.K.); zhouweixi@sicnu.edu.cn (W.Z.); hwang@stu.sicnu.edu.cn (H.W.); 2 Virtual Reality Key Laboratory of Sichuan Province, Sichuan Normal University, Chengdu 610066, China; 3 School of Information Science and Technology, Southwest Jiaotong University, Chengdu 611756, China; dr.s.zhang@ieee.org

**Keywords:** nonlinear active noise control, kernel filtered-x least mean square algorithm, error-correction learning, nonlinearity issues

## Abstract

This paper introduces a reduced Gaussian kernel filtered-x least mean square (RGKxLMS) algorithm for a nonlinear active noise control (NANC) system. This algorithm addresses the computational and storage challenges posed by the traditional kernel (i.e., KFxLMS) algorithm. Then, we analyze the mean weight behavior and computational complexity of the RGKxLMS, demonstrating its reduced complexity compared to existing kernel filtering methods and its mean stable performance. To further enhance noise reduction, we also develop the historical error correction RGKxLMS (HECRGKxLMS) algorithm, incorporating historical error information. Finally, the effectiveness of the proposed algorithms is validated, using Lorenz chaotic noise, non-stationary noise environments, and factory noise.

## 1. Introduction

Based on the superposition principle, active noise control (ANC) technology utilizes loudspeakers to produce anti-phase sound waves with equal amplitude to cancel out unwanted noise [1,2,3,4]. However, there are some nonlinearities in ANC systems, such as distortion of the output signal and saturation nonlinearity if the loudspeakers and microphones are exposed to sound pressure levels above their tolerance limits. Furthermore, the reference signal may include nonlinear components, such as chaotic systems like Logistic chaotic noise [5], Henon chaotic noise, and Lorenz chaotic noise. As a result, linear ANC systems that utilize the filtered-x least mean square (FxLMS) [6] algorithm might experience reduced effectiveness in noise reduction.

In recent decades, a number of nonlinear filters have been researched [7,8,9,10] and developed to address various challenges in nonlinear ANC (NANC) systems. Based on the Stone–Weierstrass approximation theorem, Volterra filters (VF) [11] approximate nonlinear systems by using the Volterra extension. Nevertheless, the computational complexity of the VF algorithm increases exponentially with the rise in filter order. Like the VF algorithm, the functional link artificial neural network (FLANN) is likewise a linear-in-the-parameters (LIP) network that uses various orthogonal basis functions, including Trigonometric [12], Chebyshev [13], Legendre [14], and Fourier [15]. However, FLANN filters exhibit poor noise reduction performance in highly NANC systems. To address this problem, using the reproducing kernel Hilbert space (RKHS), the kernel FxLMS (KFxLMS) algorithm, as proposed in [16] demonstrates excellent nonlinear processing capabilities. However, its memory and computational complexity increase significantly with more adaptive iterations. To curb the growth of the kernel method network, various methods have been proposed, including the random Fourier filter (RFF) [17] method, which restricts the input to a fixed-dimensional RFF space, and the Nyström (NM) [18] method, which approximates the Gram matrix of the original kernel by a low-rank matrix. For the RFF, the accuracy of the approximation kernel is influenced by both the number of fixed dimensions and the frequency parameter of the random Fourier features. The NM method necessitates prior knowledge to generate a low-rank matrix that follows the same distribution as the reference signal. In addition to traditional nonlinear filters, deep learning-based ANC systems have emerged as a powerful alternative, leveraging neural network architectures and large-scale training datasets to capture complex nonlinear noise features [19,20,21,22]. For example, ref. [23] proposed DNoiseNet, which is a deep learning-based feedback ANC system that significantly enhances noise reduction in dynamic environments. Similarly, ref. [24] introduced CsNNet, which is a lightweight model combining convolutional neural networks (CNNs), long short-term memory (LSTM), and attention mechanisms, which effectively suppresses nonlinear and highly dynamic construction noise. While these methods offer superior noise reduction compared to traditional algorithms, their reliance on substantial computational resources limits their deployment in resource-constrained environments. Therefore, there is a need for novel approaches that achieve a better trade-off between computational efficiency and noise reduction performance in NANC systems.

In this paper, we design two reduced Gaussian kernel filter-x LMS (RGKxLMS) algorithms for NANC systems. First, using a reduced Gaussian kernel proposed in [25], an RGKxLMS algorithm is presented. Compared to existing kernel FxLMS algorithms, the RGKxLMS algorithm exhibits lower computational complexity. In addition, the mean weight behavior of RGKxLMS algorithm is analyzed. On the basis of the RGKxLMS algorithm, we further introduce the HECRGKxLMS algorithm to fully utilize the previously discarded error information. In the algorithm, the historical filtering errors are collected to compensate for the filtered output at present, further enhancing the noise reduction ability in the NANC system. Finally, to assess the noise reduction capability of the proposed algorithms, a series of simulation experiments is carried out.

## 2. Review of NANC Model and KFxLMS Algorithms

**Figure 1:**
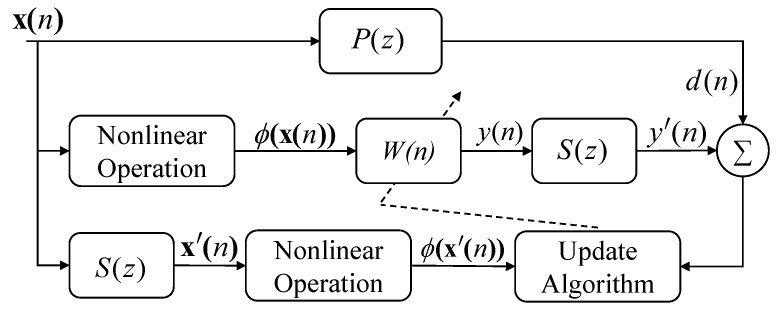
Structure diagram of NANC system.

The general NANC system block diagram is shown in Figure 1, where P(z) represents the transfer function of the primary path from noise source to error microphone, S(z) denotes transfer function of the secondary path from the loudspeaker to the error microphone, d(n) is the desired noise signal, and $x\left(n\right)={\left[\begin{array}{c} x(n),x(n-1),\ldots ,x(n-L-1) \end{array}\right]}^{T}$ denotes the noise input vector at discrete time *n*. Here, *L* refers to the filter length. By applying a nonlinear operation ϕ(·) to the input signal, the filter output is expressed as

(1) $$y\left(n\right)=w^{T}\left(n-1\right)\phi \left(x\left(n\right)\right)$$

where w(n) denotes the weight vector. The error microphone captures the residual noise e(n) as

(2) $$e\left(n\right)=d\left(n\right)-s\left(n\right)*\left[\begin{array}{c} w^{T}\left(n-1\right)\phi \left(x\left(n\right)\right) \end{array}\right]$$

where s(n) denotes the impulse response of the secondary path S(z), and * indicates the convolution operation. The weight vector w(n) in the KFxLMS algorithm is updated as:

(3) $$\begin{array}{cc} w(n) & =w\left(n-1\right)+\mu e\left(n\right)\phi (x^{\prime }\left(n\right))\\ \; & =\mu \sum_{i=1}^{n}e\left(i\right)\phi \left(x^{\prime }\left(i\right)\right) \end{array}$$

where μ represents the step size, w(0) indicates the initial weight vector, which is assumed to be zero, and $x^{\prime }\left(n\right)=\hat{s}\left(n\right)*x\left(n\right)$ with $\hat{s}\left(n\right)$ being the estimated finite impulse response filter of S(z). Substituting (3) into (1) yields the output of the filter

(4) $$y\left(n\right)=\mu \sum_{i=1}^{n-1}e\left(i\right)\phi^{T}\left(x^{\prime }\left(i\right)\right)\phi \left(x\left(n\right)\right)$$

Based on Mercer’s theorem, (4) can be described as

(5) $$y\left(n\right)=\mu \sum_{i=1}^{n-1}e\left(i\right)\kappa \left(x^{\prime }\left(i\right),x\left(n\right)\right)$$

where the Gaussian kernel function $\kappa \left(x(i),x(j)\right)=\exp\left(-{∥x(i)-x(j)∥}^{2}/\left(2\sigma^{2}\right)\right)$ is used throughout the paper and σ>0 is the kernel bandwidth.

As can be seen, (5) requires calculating the kernel function at the current moment and across all previous moments. When the number of iterations is too large, their complexity is extremely high. According to the Bochner’s theorem, the Gaussian kernel $\kappa \left(x(i),x(j)\right)$ can be expressed as

(6) $$\begin{array}{cc} \kappa \left(x(i),x(j)\right) & =E_{\omega }\left[\cos\left(\omega^{T}\left(x\left(i\right)-x\left(j\right)\right)\right)\right]\\ \; & =E_{\omega ,\theta }\left[z_{\omega ,\theta }^{T}\left(x\left(i\right)\right)z_{\omega ,\theta }\left(x\left(j\right)\right)\right] \end{array}$$

where $z_{\omega ,\theta }\left(x\left(i\right)\right)=\sqrt{2}\cos\left(\omega^{T}x\left(i\right)+\theta \right)$ represents the transformed input vector in the RFF space [26], expressed as

(7) $$z\left(x\right)=\sqrt{\frac{2}{D}}{\left[\cos\left(\omega_{1}^{T}x+\theta_{1}\right),...,\cos\left(\omega_{D}^{T}x+\theta_{D}\right)\right]}^{T}$$

In (7), $i.i.d.\;{\left\{\omega_{l}\right\}}_{l=1}^{D}$ are sampled from the multivariate Gaussian distribution $N(0,\sigma^{-2}I_{L})$ with $I_{L}$ representing the unit matrix, *L* denotes the dimension of the input signal, and $i.i.d.{\left\{\theta_{l}\right\}}_{l=1}^{D}$ are sampled from a uniform distribution U[0,2π], where *D* denotes the dimension of the RFF space.

Although the use of this transformation vector (7) enables the random Fourier-based FxLMS (RFFxLMS) algorithm to effectively reduce the input growth space of the KFxLMS algorithm, the frequency ω is obtained via random sampling, which could affect the accuracy of the kernel adaptive filtering algorithm’s approximation. In addition, a higher RFF space dimension *D* can improve the accuracy of the approximation by capturing more details of the input signal. However, increasing dimension *D* would impose a computational burden on a real-time ANC system.

## 3. Proposed Algorithms

### 3.1. Proposed RGKxLMS Algorithm

In Ref. [25], for a vector $x={\left[x_{1},x_{2},\cdots ,x_{\ell }\right]}^{T}$, the reduced Gaussian kernel function was developed, via using the following approximate feature mapping function:

(8) $$\begin{array}{c} \varphi \left(x\right)={\left[\varphi_{0}^{T}\left(x\right),\varphi_{1}^{T}\left(x\right),\cdots ,\varphi_{m}^{T}\left(x\right),\cdots ,\varphi_{p}^{T}\left(x\right)\right]}^{T} \end{array}$$

where *p* is an order and

(9) $$\begin{array}{cc} \varphi_{m}\left(x\right)=\sqrt{\frac{1}{m!\sigma^{2m}}} & [x_{1}\exp\left(-\frac{x_{1}^{2}}{2\sigma^{2}}\right),x_{2}\exp\left(-\frac{x_{2}^{2}}{2\sigma^{2}}\right),\cdots ,x_{\ell }\exp\left(-\frac{x_{\ell }^{2}}{2\sigma^{2}}\right){]}^{T} \end{array}$$

Based on (8), using the input vector $x\left(n\right)={\left[\begin{array}{c} x(n),x(n-1),\ldots ,x(n-L-1) \end{array}\right]}^{T}$ at discrete time *n*, the output of our NANC filter can be written as

(10) $$y\left(n\right)=\sum_{i=0}^{p}w_{i}^{T}\left(n-1\right)\varphi_{i}\left(x\left(n\right)\right)=w^{T}\left(n-1\right)\varphi \left(x\left(n\right)\right)$$

where the vector $w\left(n\right)={\left[w_{0}^{T}\left(n\right),w_{1}^{T}\left(n\right),\cdots ,w_{p}^{T}\left(n\right)\right]}^{T}$ represents the filter weight estimation.

Define a residual error e(n) as

(11) $$e\left(n\right)=d\left(n\right)-s\left(n\right)*[w^{T}\left(n-1\right)\varphi \left(x\left(n\right)\right)]$$

Using stochastic gradient update to minimize $E\{e^{2}\left(n\right)\}$, we obtain the updated formula of the RGKxLMS algorithm:

(12) $$w\left(n\right)=w\left(n-1\right)+\mu e\left(n\right)\varphi (x^{\prime }\left(n\right))$$

where $\varphi^{\prime }\left(x\left(n\right)\right)=\hat{s}\left(n\right)*\varphi \left(x\left(n\right)\right)$ represents the filtered input vector.

### 3.2. Proposed HECRGKxLMS Algorithm

Traditional NANC systems disregard errors after updating the weight vector. It should be noted, however, that these discarded errors may provide valuable information for reducing residual noise [27]. Using (11) at the current moment *n*, we can obtain the residual signal e(n) at the error microphone. If a historical signal $\varphi (x\left(t^{o}\right))$ ($t^{o}\in \left\{1,2,\cdots ,n-1\right\}$) identical to φ(x(n)) can be found for the RGKxLMS algorithm, the current output value can be corrected by performing the following:

(13) $$e_{c}\left(n\right)=d\left(n\right)-\left(s\left(n\right)*y\left(n\right)+e\left(t^{o}\right)\right)$$

where the historical error signal $e(t^{o})$ corresponds to $\varphi (x\left(t^{o}\right))$ and is computed as $e\left(t^{o}\right)=d\left(t^{o}\right)-s\left(n\right)*\left[\begin{array}{c} w^{T}\left(n-1\right)\varphi \left(x\left(t^{o}\right)\right) \end{array}\right]$.

Inspired by nearest neighbor estimation (NNE) [28], to find the similar historical signal $\varphi (x\left(t^{o}\right))$ with low complexity, we use Manhattan distance:

(14) $$t^{o}=\arg\min_{1\leq t\leq n-1}\left|\varphi \left(x^{\prime }\left(n\right)\right)-\varphi \left(x^{\prime }\left(t\right)\right)\right|$$

Then, to achieve the corrected error in (13), the inverse of the secondary path $s^{-1}\left(n\right)$ is employed, then the output of corrected filter $y_{c}\left(n\right)$ is

(15) $$y_{c}\left(n\right)=y\left(n\right)+s^{-1}\left(n\right)*e\left(t^{o}\right)$$

**Remark** **1.** *In contrast with linear active noise [1,4], our work uses a kernel-based nonlinear extension (8) and historical error correction (15), which makes it more suitable for nonlinear active noise control environments.*

## 4. Performance Analysis

In this section, we analyze the stability and computational complexity of the designed algorithm.

### 4.1. Mean Behavior

In this subsection, we analyze the mean behavior of the RGKxLMS algorithm, with the following assumptions:

A1: The reference signal x(n) is derived from a stationary random process with a zero mean.

A2: The weight vector w(n) is independent of φ(x(n)).

As illustrated in Figure 1, the expanded input signal is driven by the controller W(n), resulting in the loudspeaker output y(n), which is subsequently passed through the physical secondary path S(z) to yield the anti-noise signal $y^{\prime }\left(n\right)$. Assuming that secondary path is accurately estimated, i.e., $S\left(z\right)=\hat{S}\left(z\right)$, and defining $s\left(n\right)=\hat{s}\left(n\right)={\left[s\left(n\right),s\left(n-1\right),\cdots ,s\left(n-F+1\right)\right]}^{T}$, this process can be mathematically expressed as:

(16) $$\begin{array}{cc} y^{\prime }\left(n\right) & =\sum_{j=0}^{F-1}y\left(n-j\right)s\left(n-j\right))=\sum_{j=0}^{F-1}w^{T}\left(n-j\right)\varphi \left(x\left(n-j\right)\right)s\left(n-j\right)\\ \; & =W^{T}\left(n\right)X_{f}\left(n\right) \end{array}$$

where $X_{f}\left(n\right)$ is the filtered input augmented vector, and W(n) is the augmented weight vector, defined as:

(17) $$X_{f}\left(n\right)=\left[\begin{array}{c} s(n))\;\varphi (x(n))\\ s(n-1)\varphi (x(n-1))\\ \vdots \\ s(n-F+1)\varphi (x(n-F+1)) \end{array}\right],\;W\left(n\right)=\left[\begin{array}{c} w(n)\\ w(n-1)\\ \vdots \\ w(n-F+1) \end{array}\right]$$

Define $\hat{w}\left(n\right)=w\left(n\right)-w_{\mathrm{opt}}$. We consider that $P\left(z\right)/S\left(z\right)=W_{\mathrm{opt}}$, and with the S(z) known [29], the desired signal is given by

(18) $$\begin{array}{cc} d(n) & =\varphi \left(x\left(n\right)\right)*p\left(n\right)=\varphi^{T}\left(x\left(n\right)\right)*\left(w_{\mathrm{opt}}*s\left(n\right)\right)\\ \; & \approx W_{\mathrm{opt}}^{T}X_{f}\left(n\right) \end{array}$$

where $W_{\mathrm{opt}}={\left[w_{\mathrm{opt}}^{T},w_{\mathrm{opt}}^{T},\cdots ,w_{\mathrm{opt}}^{T}\right]}^{T}$ is an augmented vector of $w_{\mathrm{opt}}$. Then, rewriting (12), we obtain

(19) $$\begin{array}{cc} \hat{w}\left(n+1\right) & =\hat{w}\left(n\right)\;-\;\mu \left(W_{\mathrm{opt}}^{T}X_{f}\left(n\right)\;-\;W^{T}\left(n\right)X_{f}\left(n\right)\right)s\left(n\right)X^{T}\left(n\right)\\ \; & =\hat{w}\left(n\right)+\mu A\left(n\right)\hat{W}\left(n\right) \end{array}$$

where $A\left(n\right)=X\left(n\right)s\left(n\right)X_{f}{\left(n\right)}^{T}$, X(n)=[φ(x(n)),φ(x(n−1)),…,φ(x(n−F+1))] contains the past *F* expanded input vectors matrix. By employing the form of augmented vector $\hat{W}\left(n\right)=W_{\mathrm{opt}}-W\left(n\right)$ and defining the transfer matrix, we derive the following relationship:

(20) $$B=\left[\begin{array}{ccccc} I_{L} & 0_{L} & 0_{L} & \cdots & 0_{L}\\ I_{L} & 0_{L} & 0_{L} & \cdots & 0_{L}\\ 0_{L} & I_{L} & 0_{L} & \cdots & 0_{L}\\ \vdots & \vdots & \ddots & \ddots & \vdots \\ 0_{L} & 0_{L} & \cdots & I_{L} & 0_{L} \end{array}\right]\;p=\left[\begin{array}{c} I_{L}\\ 0_{L}\\ 0_{L}\\ \vdots \\ 0_{L} \end{array}\right]$$

we have

(21) $$\hat{W}\left(n+1\right)=B\hat{W}\left(n\right)+p\left(\hat{w}\left(n+1\right)-\hat{w}\left(n\right)\right)$$

where the B with dimension FL×FL ensures an ordered shift of the elements in $\hat{W}$ and the p with dimension FL×L ensures the accumulation at the current moment. Rewriting (19) using the structure of (21) and taking expectations, we obtain

(22) $$E\left[\hat{W}\left(n+1\right)\right]=E\left[Z\left(n\right)\right]E\left[\hat{W}\left(n\right)\right]$$

where Z(n)=B−μpA(n). Therefore, the RGKxLMS algorithm is mean-stable when the spectral radius of the matrix E[Z(n)] is less than 1. Since the HECRGKxLMS algorithm is an enhanced version of the RGKxLMS algorithm, the compensation term $e_{c}\left(n\right)$ derived from historical error correction does not impact the weight update process. Consequently, the average weight behavior described in (22) remains applicable to the HECRGKxLMS algorithm. Substituting (16), (18) into (12), we obtain the error signal

(23) $$\begin{array}{cc} e_{c}\left(n\right) & =W_{\mathrm{opt}}^{T}X_{f}\left(n\right)-y^{\prime }\left(n\right)-e\left(t^{0}\right)\\ \; & =W_{\mathrm{opt}}^{T}X_{f}\left(n\right)-W^{T}\left(n\right)X_{f}\left(n\right)-W_{\mathrm{opt}}^{T}X_{f}\left(t^{0}\right)-W^{T}\left(n\right)X_{f}\left(t^{0}\right)\\ \; & ={\hat{W}}^{T}\left(n\right)\left(X_{f}\left(n\right)-X_{f}\left(t^{0}\right)\right) \end{array}$$

Taking the expectation of both sides of the above equation, we obtain

(24) $$\begin{array}{cc} E[e_{c}\left(n\right)] & =E\left[{\hat{W}}^{T}\left(n\right)\left(X_{f}\left(n\right)-X_{f}\left(t^{0}\right)\right)\right]\\ \; & =E\left[{\hat{W}}^{T}\left(n\right)\right]E\left[(X_{f}\left(n\right)-X_{f}\left(t^{0}\right))\right] \end{array}$$

It can be observed from (24) that $E[e_{c}\left(n\right)]$ depends on $E\left[(X_{f}\left(n\right)-X_{f}\left(t^{0}\right))\right]$ at the steady state. Consequently, if a value $X_{f}\left(t^{0}\right)$ can be identified that is sufficiently close to $X_{f}\left(n\right)$, the error signal $e_{c}\left(n\right)$ can be significantly minimized. In addition, (9) expands the input vector to capture nonlinear components of the NANC system to achieve better performance than the linear ErFxLMS algorithm.

### 4.2. Computational Complexity

We provide a summary of the computational complexity of several nonlinear algorithms, including VFxLMS [11], FsLMS [30], KFxLMS [16], RFFxLMS [17], and RGKxLMS, in Table 1, where *M* represents the expansion order of the VFxLMS and FsLMS algorithms, *p* is the order of RGKxLMS algorithm, and the linear convolution requires *F* multiplications and F−1 additions. The complexity of the HECRGKxLMS algorithm is not included in this table because its complexity is influenced not only by the algorithm’s internal operations but also by the linear search component. The number of multiplications and additions required by the KFxLMS algorithm in Table 1 increases linearly with the number of adaptive iterations. Since the RGKxLMS algorithm provides a method for explicit kernel mapping, the computational load required for each iteration remains constant. Furthermore, in comparison to other nonlinear algorithms listed in the table, the RGKxLMS algorithm requires fewer nonlinear operations.

## 5. Simulation Results

This section compares the performance of the proposed RGKxLMS and HECRGKxLMS algorithms with VFxLMS, FsLMS, RFFxLMS, KFxLMS, and ErFxLMS [31] on NANC systems. Simulation curves were generated by averaging the results of 50 Monte Carlo simulations. The averaged noise reduction (ANR) is employed to evaluate performance, which is defined as

(25) $$\mathrm{ANR}\left(n\right)=20\log\frac{A_{e}\left(n\right)}{A_{d}\left(n\right)}$$

where $A_{e}\left(n\right)=\xi A_{e}\left(n-1\right)+\left(1-\xi \right)\left|e_{n}\right|$ and $A_{d}\left(n\right)=\xi A_{d}\left(n-1\right)+\left(1-\xi \right)\left|d_{n}\right|$ and the forgetting factor is ξ=0.999. The noise cancellation is disturbed by a third-order polynomial, and the desired signal is given by

(26) $$d\left(n\right)=b\left(n-2\right)+0.8b^{2}\left(n-2\right)-0.4b^{3}\left(n-1\right)$$

where b(n)=x(n)∗r(n) where r(n) represents the transfer function of P(z). We assume the secondary path is modeled offline, where $S\left(z\right)=\hat{S}\left(z\right)$.

### 5.1. Mean Weight Convergence

**Figure 2:**
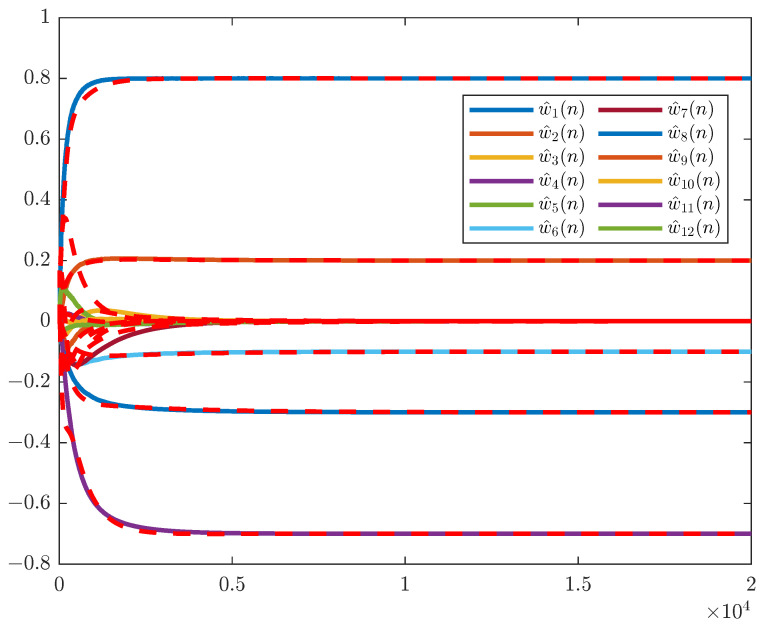
Mean behavior of the RGKxLMS algorithm.

In this subsection, we simulate the weight convergence curves of RGKxLMS algorithm and use (22) to obtain the theoretical weight. The primary path is modeled as $P\left(z\right)=z^{-3}-0.3z^{-4}+0.2z^{-5}$. The secondary path considered offline modeling with minimum phase $S\left(z\right)=z^{-2}+0.5z^{-3}$. In Figure 2, we use a white noise input signal, where the step size is set to μ=0.02, the filter length is L=3, and the nonlinear expansion order is p=3. The result shows that the weight behavior matches the theoretical analysis.

### 5.2. Comparison of Compensation Ability

**Figure 3:**
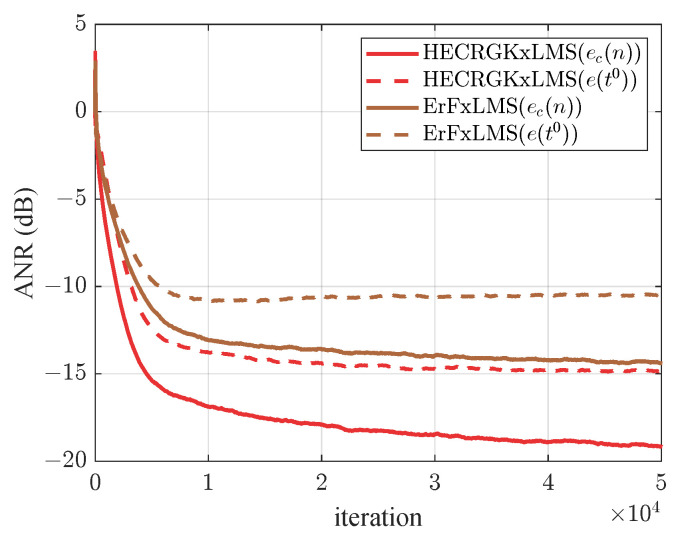
Comparison of ANR curves for two different types of errors for HECRGKxLMS and ErFxLMS.

In this subsection, to illustrate the advantages of the HECRGKxLMS algorithm in NANC systems, we used Gaussian white noise with variance 0.3 and zero mean as a reference signal, and the nonlinearities of the primary path were modeled by (26). Figure 3 compares the ANRs of the residual errors between the HECRGKxLMS and ErFxLMS algorithms, where $e_{c}\left(n\right)$ is measured by the error microphone and $e(t^{o})$ is searched by the NNE. From Figure 3, it can be observed that the proposed HECRGKxLMS algorithm possesses a lower ANR than the ErFxLMS algorithm in the presence of strong nonlinearities in the primary path. Since the RGKxLMS algorithm demonstrates excellent capability in solving nonlinear problems, the HECRGKxLMS algorithm with historical error correction exhibits superior performance.

### 5.3. Lorenz Chaotic Noise

**Figure 4:**
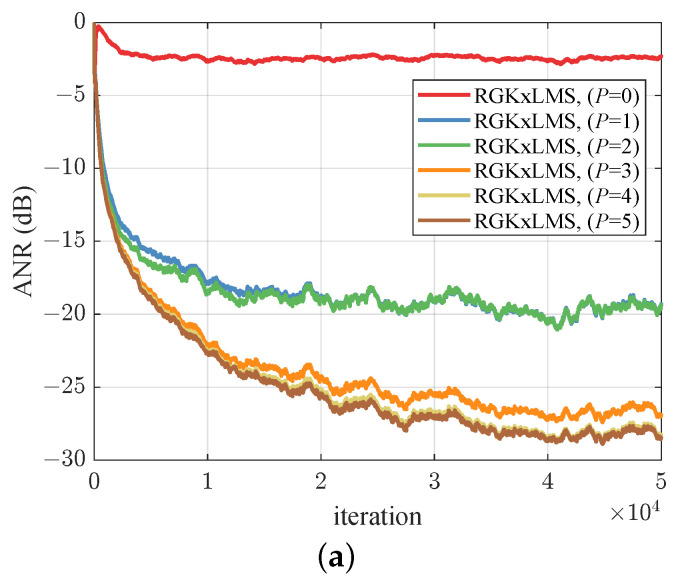

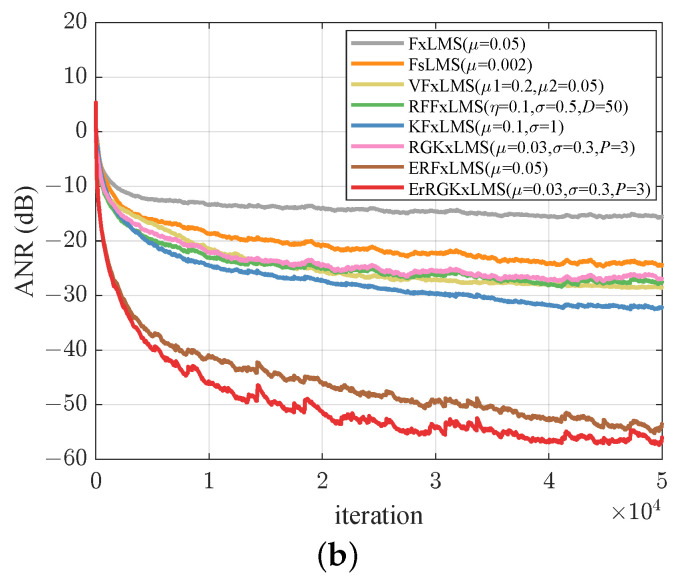
ANR learning curves for Lorenz chaotic noise. (**a**) RGKxLMS algorithm with different expanded order *p*. (**b**) Compared curves of ANR performance.

We consider a Lorenz chaotic system [5], which is generated by solving the following system of ternary first-order ordinary differential equations:

(27) $$\left\{\begin{array}{c} \frac{dx}{dt}=-\epsilon x+yz\\ \frac{dy}{dt}=\gamma \left(z-y\right)\\ \frac{dz}{dt}=-xy+\delta y-z \end{array}\right.$$

where ϵ=3/8, γ=10, and δ=28. Given that the model generates three variables, we utilized a normalized reference signal of 50,000 samples along the *x*-axis. This sequence was processed to achieve a mean of 0 and a variance of 1. Figure 4a illustrates that as the order of the expansion increases, the noise reduction performance improves; however, the extent of this improvement diminishes progressively after p>3. Consequently, in all subsequent experiments, we have chosen p=3. To maintain fairness, we have standardized the length of the extended input vectors across all algorithms. Specifically, the VFxLMS and FsLMS algorithms are set to an order of M=2, and the RFF space dimension is fixed at D=50. In Figure 4b, the RGKxLMS, RFFxLMS, and VFxLMS algorithms exhibit similar performance. The HECRGKxLMS algorithm exceeds the RGKxLMS algorithm due to the historical error correction. This demonstrates that error compensation can improve the noise reduction capabilities of the algorithms.

### 5.4. Non-Stationary Noise Environments

**Figure 5:**
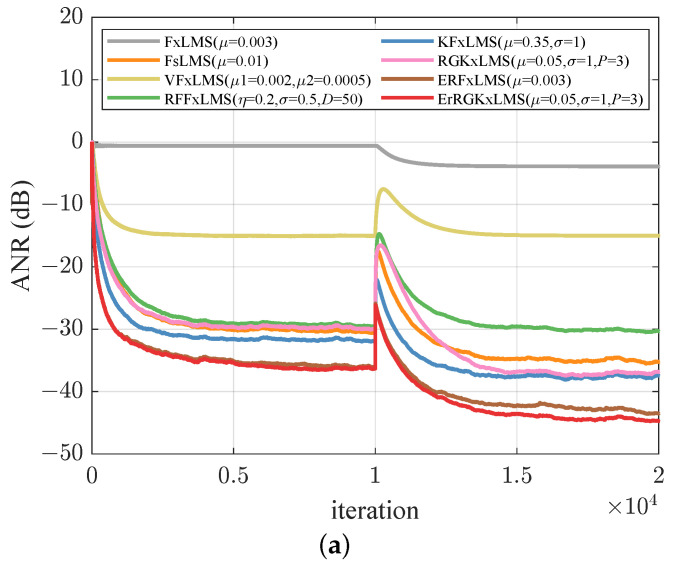

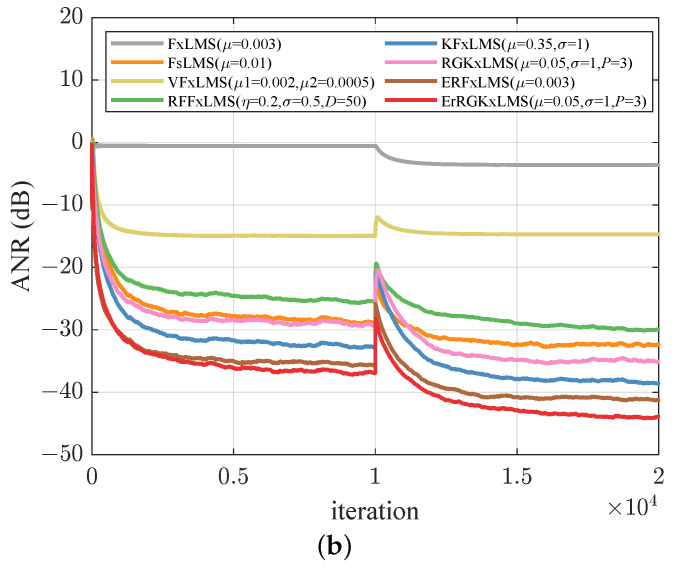
Compared curves of ANR performance under non-stationary reference noise with different secondary paths: (**a**) minimum phase, (**b**) non-minimum phase.

In this section, we simulate a sinusoidal wave with a sudden frequency change to verify the dynamic characteristics of the proposed algorithm, which is described as

(28) $$x\left(n\right)=\sqrt{2}\sin\left(\frac{2\pi \times f_{s}\times n}{8000}\right)+g\left(n\right)$$

where the sampling rate is set to 8000 samples/s, and g(n) represents white Gaussian noise with a signal-to-noise ratio (SNR) of 40 dB. In the initial stage, we set the frequency to $f_{s}=500$ Hz. After 10,000 iterations, the frequency is changed to $f_{s}=3000$ Hz. We also consider a system with a non-minimum phase, where the transfer function is denoted as $S\left(z\right)=z^{-2}+1.5z^{-3}-z^{-4}$. From Figure 5a,b, the FxLMS algorithm demonstrates poor noise reduction capability when strong nonlinearities occur in the primary path. In both cases, following a frequency mutation of the sinusoidal wave, the steady-state ANR of the RGKxLMS algorithm is comparable to that of KFxLMS. Using a historical error correction strategy allows the HECRGKxLMS to outperform other algorithms.

### 5.5. Real Recorded Factory Floor Noise Control

**Figure 6:**
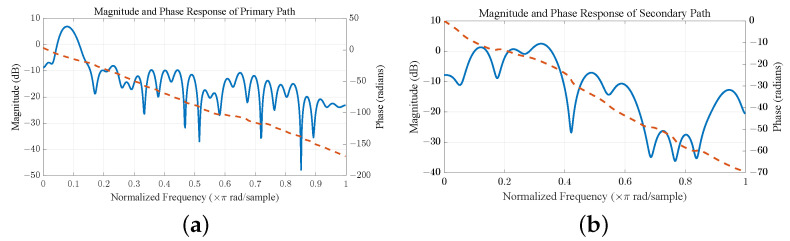
(**a**) Primary path. (**b**) Secondary path.

**Figure 7:**
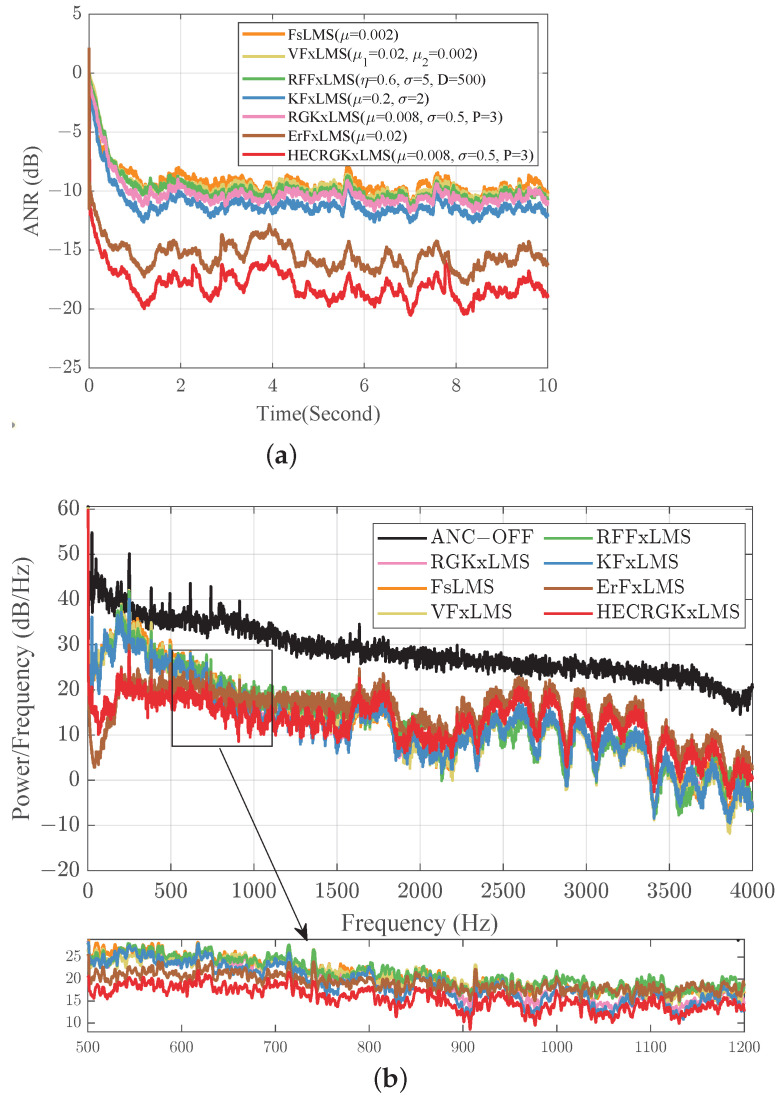
(**a**) ANR curves of different nonlinear algorithms. (**b**) PSD comparison of residual noise under factory floor noise.

In this experiment, the factory floor noise provided from http://spib.linse.ufsc.br/database.html (accessed on 1 August 2024) is used as the reference signal, which is down-sampled to an 8 kHz sampling rate, and a 16-bit digital-to-analog converter is used to capture the first 10 s [32]. The primary and secondary paths are represented by FIR filters with lengths of 64 and 32 taps, respectively [1], with their magnitude and phase responses depicted in Figure 6a,b. We also still use third-order polynomials (24), and the length of the weight vector is L=100. Figure 7a illustrates the noise reduction capability of various nonlinear algorithms. The average ANRs of the last 1s are −9.60 dB for FsLMS; −10.08 dB for VFxLMS; −10.20 dB for RFFxLMS; −11.46 dB for KFxLMS; −10.46 dB for RGKxLMS; −15.52 dB for ErFxLMS; and −18.21 dB for HECRGKxLMS. The HECRGKxLMS algorithm achieves the best overall performance, significantly outperforming other methods. Figure 7 shows the power spectral density (PSD) across the 0–4000 Hz range (top) and a zoomed-in view of the 500–1200 Hz range (bottom). The RGKxLMS algorithm, designed as a low-complexity alternative to KFxLMS, shows slightly reduced performance in the 500–900 Hz range but delivers comparable results in the 900–1200 Hz range. This highlights the effectiveness of the proposed approximation in balancing computational efficiency and noise reduction performance. Additionally, the proposed HECRGKxLMS algorithm achieves a remarkable noise reduction of −18.69 dB within the 500–1200 Hz range, outperforming all other algorithms. Although the waterbed effect leads to a reduction in noise cancellation performance at higher frequencies [4], the HECRGKxLMS algorithm overall demonstrates superior noise reduction across all frequency bands. This is particularly relevant for ANC systems, which primarily target low-frequency noise due to the limitations of passive noise isolation methods [1]. The proposed algorithm ensures substantial noise reduction in low-frequency regions while maintaining robust performance across other bands, significantly enhancing the overall effectiveness of the ANC system.

## 6. Conclusions

In real-time active noise control (ANC) systems, it is essential to develop algorithms with low computational complexity. This paper introduces the RGKxLMS algorithm as a low-complexity alternative to the KFxLMS algorithm and analyzes its mean weight behavior. To further enhance performance, a historical error correction strategy is incorporated, which significantly improves noise reduction but at the cost of increased computational complexity. Moreover, due to the waterbed effect, improvements in noise suppression within certain frequency ranges may lead to reduced performance in other ranges, resulting in persistent noise amplification at specific frequencies. Addressing this trade-off by dynamically adjusting the affected frequency bands and optimizing the linear search in (14) will be crucial for future research. Additionally, exploring the integration of the historical error correction mechanism into deep learning-based ANC systems could offer promising improvements. Another important direction for future work is to address loudspeaker output saturation under high input signals, which is a limitation not considered in this study.

## Figures and Tables

**Table 1:** Computational complexity of different algorithms.

Algorithm	Addition	Multiplication	Nonlinear Operation
FsLMS	4L(2M−1)+2F+1	2L(2M−1)+F−1	2ML
VFxLMS	$2\frac{(L+M)!}{L!M!}+\frac{(L+M-1)!}{(L-1)!M!}+2F$	$2\frac{(L+M)!}{L!M!}+F-3$	−
RFFxLMS	D(L+2)+2F+2	D(L+2)+F−1	*D*
KFxLMS	n(L+F+2)+F+1	n(2L−1)+F	n−1
RGKxLMS	4L(p+1)+2F+L+2	2L(p+1)+F+L−2	*L*

## Data Availability

No new data were created or analyzed in this study. Data sharing is not applicable to this article.
